# Copper nanofiber-networked cobalt oxide composites for high performance Li-ion batteries

**DOI:** 10.1186/1556-276X-6-292

**Published:** 2011-04-05

**Authors:** Sang Hoon Nam, Yong Seok Kim, Hee-Sang Shim, Jong Guk Kim, Won Bae Kim

**Affiliations:** 1 School of Materials Science and Engineering, Gwangju Institute of Science and Technology (GIST), 261 Chemdan-gwagiro, Buk-gu, Gwangju 500-712, South Korea; 2 Research Institute for Solar and Sustainable Energies (RISE), Gwangju Institute of Science and Technology (GIST), 261 Chemdan-gwagiro, Buk-gu, Gwangju 500-712, South Korea

## Abstract

We prepared a composite electrode structure consisting of copper nanofiber-networked cobalt oxide (CuNFs@CoO*_x_*). The copper nanofibers (CuNFs) were fabricated on a substrate with formation of a network structure, which may have potential for improving electron percolation and retarding film deformation during the discharging/charging process over the electroactive cobalt oxide. Compared to bare CoO*_x_*thin-film (CoO*_x_*TF) electrodes, the CuNFs@CoO*_x_*electrodes exhibited a significant enhancement of rate performance by at least six-fold at an input current density of 3C-rate. Such enhanced Li-ion storage performance may be associated with modified electrode structure at the nanoscale, improved charge transfer, and facile stress relaxation from the embedded CuNF network. Consequently, the CuNFs@CoO*_x_*composite structure demonstrated here can be used as a promising high-performance electrode for Li-ion batteries.

## Introduction

Cobalt oxide (CoO*_x_*) is a high-capacity electrode material for Li-ion batteries with a theoretical capacity of at least two times greater than that of graphite (*ca*. 370 mAh g^-1^) [1]. However, the cobalt oxides show large irreversible capacity and poor cycling performance caused by Li-alloying, agglomeration or growth of passivation layers [1]. In addition, severe volume expansion during discharge/charge process accelerates fading of the capacity, and electrical contact between the electrode material and current collector eventually fails. To overcome these problems, several strategies that employ a secondary material [2], a chemically or physically prepared surface coating [3], size optimization [4,5], and fabrication of a nanostructure [6] have been reported. These approaches generally provide a facile electrochemical reaction route, high conductivity, and structural stability. In particular, nanostuctured electrode materials are expected to be well-suited for next-generation Li-ion batteries due to their substantially increased reaction area and facilitated charge carrier transport through shortened Li-ion diffusion paths [7]. For example, Kim et al. [8] proposed a core-shell nanorod array electrode, which consists of a metallic conducting core with a vanadium oxide (VO*_x_*) shell layer. Such highly conducting core-embedded nanostructure was capable of enhancing the electrochemical properties of the VO*_x_*electrodes even though the electroactive materials have high electrical resistance. In addition, incorporation of metal into active material was found to increase the charge transfer in electrode materials along with facilitated Li-ion diffusion [9]. Therefore, it is expected that the incorporation of highly conducting metal nanowires into cobalt oxide materials would be a promising way to increase electrical conductivity and mitigate the particle agglomeration of the cobalt oxide during Li-ion insertion/extraction.

In this report, we prepared cobalt oxide electrode that is composited with copper nanofiber network, and demonstrated that such embedded nanostructure is able to enhance electrical conductivity and mechanical stability for the CoO*_x_*electrode during repeated cyclings.

## Experimental

### Fabrication of Cu nanofiber-embedded cobalt oxide composites

The composite nanostructure of copper nanofiber-networked cobalt oxide (CuNFs@CoO*_x_*) was prepared by using an electrospinning process to produce the copper nanofibers and followed by a radio frequency magnetron sputtering (RF sputtering) to deposit the CoO*_x_*materials. The electrospinning solution was prepared by mixing copper(II) chloride dihydrate (CuCl_2 _2H_2_O, Sigma-Aldrich, Saint Louis, USA), methanol, and polyvinylpyrrolidone (PVP; *M*_w _= 1,300,000 g mol^-1^, Sigma-Aldrich, Saint Louis, USA). The solution was then immediately loaded into a syringe, which was attached to a 23-gauge stainless steel needle. A 10-kV electric field was applied between the needle tip and a grounded stainless steel disc at a distance of 10 cm. The stainless steel substrate was mechanically polished before use with a sandpaper and diamond paste (ca. 0.3 μm) until a mirror-like surface was obtained. Subsequently, the collected CuCl_2_/PVP composite on the substrate was heated at 300°C for 3 h in air. To obtain the metallic CuNFs, a reduction treatment was performed at 200°C in H_2 _atmosphere at a flow rate of 60 sccm. Next, CoO*_x_*was deposited onto the Cu nanofibers-formed substrate via RF sputtering with an cobalt oxide target under an inert Ar gas atmosphere at a working pressure of 1 × 10^-3 ^Torr. The deposition thickness of the CoO*_x_*was controlled to ca. 100 nm. The mass ratio of the deposited CuNFs and CoO*_x_*was measured to be 2:3 using a micro-balance (Sartorius, M3P). The mass of the electrodes was controlled to have the similar quantity (ca. 0.125 mg) of CoO*_x_*as the active material.

### Characterization

The microstructures were characterized by field emission scanning electron microscopy (FESEM, Hitachi S-4700) and x-ray diffraction (XRD, Rigaku Ru-200B). To measure the thickness and investigate the cross section, the electrodes were deposited onto Si substrates instead of stainless steel substrates. The composition of the deposited CoO*_x_*was characterized by x-ray photoelectron spectroscopy (XPS, VG Multilab 2000) with a monochromic Al K_α _x-ray source (*E *= 1486.6 eV). Data processing was performed using the Avantage 4.54 software program. The background was corrected using the Shirley method, and the binding energy of the C 1*s *peak from the support at 284.5 eV was taken as an internal standard.

### Electrochemical measurements

The electrochemical tests were performed using a two-electrode system fabricated with the prepared materials for the working electrode and metallic Li for the counter electrode in an Ar-circulating glove box. A 1-M LiPF_6 _solution in a 1:1 volume mixture of ethylene carbonate and diethyl carbonate was used as the electrolyte. The galvanostatic discharge/charge mode at various C-rates from 0.15 to 3C was conducted with a potential window of 2.5 to 0.01 V (vs. Li/Li^+^) using a battery cycler (WonA tech, WBCS3000). 0.15C rate corresponds to a current rate of 0.135 A g^-1 ^of Co_3_O_4_, in which the theoretically complete discharge could be achieved in 6.7 h, and 3C rate corresponds to 2.7 A g^-1^. The AC impedance measurement was performed using a Solartron 1260 frequency response analyzer. An amplitude voltage of 5 mV was applied over the frequency range from 100 kHz to 10 mHz.

## Results and discussion

### Morphology and microstructure

**Figure 1:**
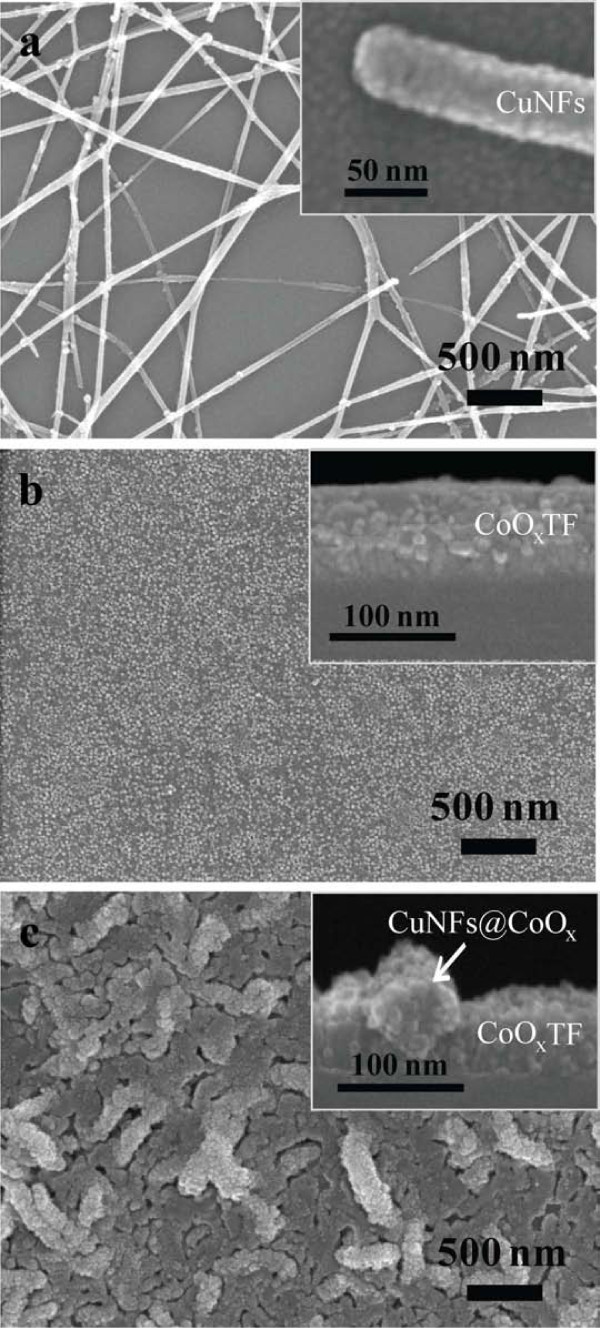
**FESEM images**. **(a) **Horizontally layered CuNFs (the inset shows a highly magnified image of the prepared nanofiber); **(b) **CoO*_x_*TF prepared by RF-sputtering (the inset shows the deposited thin-film thickness); **(c) **the composite structure of CuNFs@CoO*_x_*(the inset shows the cross-sectional view of the nanofiber and thin-film, respectively).

Figure 1a shows that the prepared CuNFs were deposited on the Si substrate and they have an average diameter of ca. 50 ± 20 nm. The surface morphology of the individual CuNF can be observed in the inset figure of Figure 1a. Figure 1b shows the bare CoO*_x_*film structure. On the other hand, Figure 1c represents a combined morphology of both nanostructures of the one-dimensional CuNFs and the CoO*_x_*. The CuNFs@CoO*_x_*has a rough surface compared to the bare CoO*_x_*TF, which may be ascribed to the presence of the CuNF network on the substrate. The sputtered CoO*_x_*was deposited not only on the substrate but also on the surface of CuNFs. After CoO*_x_*deposition, all the CuNFs seem to be covered by the CoO*_x_*layer.

**Figure 2:**
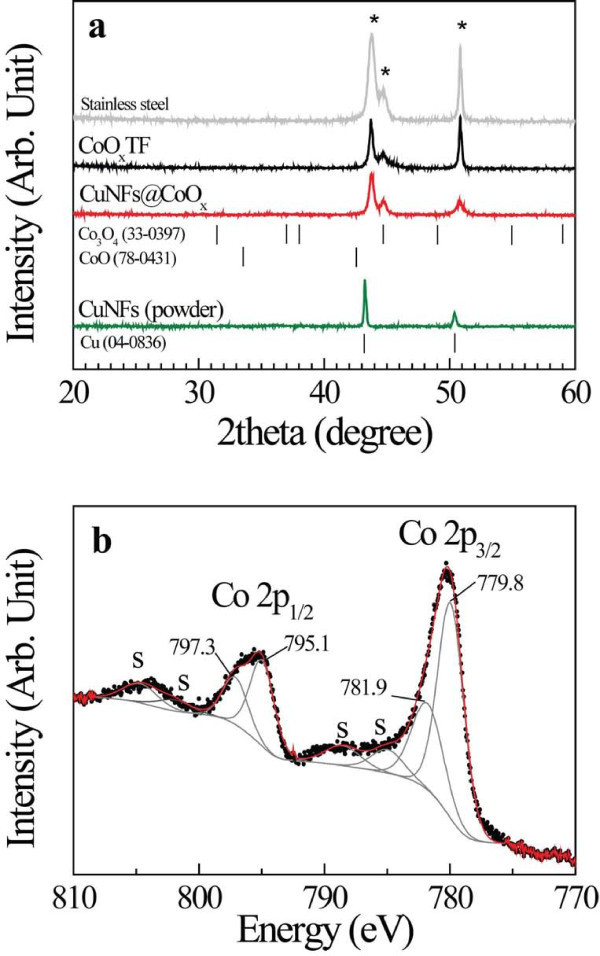
**Microstructural properties**. **(a) **x-ray diffraction patterns of the CuNFs, CoO*_x_*TF, and CuNFs@CoO*_x_*on a stainless steel disc. For the case of CuNFs, we loaded a large amount of CuNFs to acquire a significant signal. The asterisk mark can be indexed to the stainless steel substrate; **(b) **x-ray photoelectron spectrum for Co 2p_3/2 _and 2p_1/2 _of the sputtered CoO*_x_*.

The crystalline information and chemical composition of deposited electrode materials have been elucidated by XRD and XPS. Figure 2a compares the XRD patterns of CuNFs, CoO*_x_*TF, and CuNFs@CoO*_x_*, respectively. The sputtered CoO*_x_*on the stainless steel substrate indicated an amorphous nature because the diffraction pattern did not show any crystalline peaks from cobalt oxides, except the well-defined peaks from the stainless steel disc. The amorphous phase typically exhibits a high capacity and good cycling performance due to the internal stress relaxation generated by discharge/charge process [10]. The characteristic peaks of the CuNFs were observed at the expected diffraction angles from the Cu(111) and Cu(200) planes [JCPDS 04-0836]. In order to confirm the chemical state of deposited CoO*_x_*, XPS analysis was employed. In Figure 2b, the deposited CoO*_x_*gives two main peaks at 779.8 and 795.1 eV due to the Co 2p_3/2 _and Co 2p_1/2_, respectively, together with two satellite peaks at 788.6 and 803.7 eV. The peak splitting between Co 2p_3/2 _and Co 2p_1/2_, corresponding to the spin-orbit doublet of the Co 2p, is ca. 15.3 eV, and the weak and broad satellite peak of the Co 2p_3/2 _appears at ca. 9 eV higher than the main peak. Such a low-intense satellite can be considered as an indication of the Co_3_O_4 _phase [11,12], while the satellite peak of the CoO phase is relatively more intense (ca. 30% of the total Co 2p_3/2 _signal) [13]. These results indicate that the sputtered CoO*_x_*is dominantly of Co_3_O_4 _phase, which is consistent with its electrochemical properties, as will be discussed later.

### Electrochemical properties

**Figure 3:**
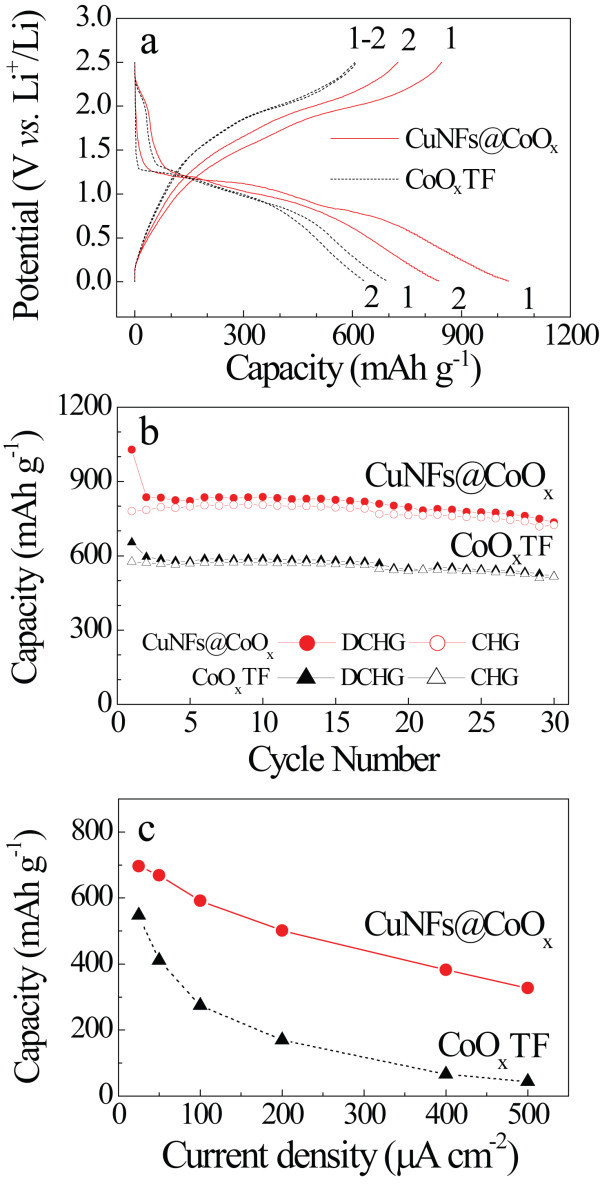
**Galvanostatic mode at 0.15C**. The potential curves of **(a) **the initial cycling profiles; **(b) **the specific capacity with cycling number; **(c) **the rate performance test for various C-rates of 0.15 to 3C.

To investigate the influence of CuNFs on the Li ions storage performance of the CoO*_x_*, we conducted galvanostatic discharge/charge processes. Figure 3a shows the first and second discharge/charge voltage profiles at a constant 0.15C between the voltages of 2.5 and 0.01 V (vs. Li^+^/Li). Both CoO*_x_*TF and CuNFs@CoO*_x_*exhibit the plateau around 1.0 V in the first discharge curve. This is associated with the following electrochemical reaction [14] of Co_3_O_4 _+ 8Li^+ ^+ 8e^- ^→ 4Li_2_O + 3Co. The CuNFs@CoO*_x _*seems to have a little bit larger irreversible capacity of ca. 240 mAh g^-1 ^compared to the bare CoO*_x_*TF, which could be caused by the enlarged contact area between the electrolyte and electrode material [7]. Although the CuNFs@CoO*_x_*electrode indicated a conversion profile similar to that of the CoO*_x_*TF, the capacity was ca. 30% higher than that of the bare CoO*_x_*TF, as shown in Figure 3b. The highly rugged microstructure of CuNFs@CoO*_x_*might be responsible for the increased reaction sites along the CuNF network, making the electrochemical reaction more efficient with Li ions, because the electrochemical performance can be dependent on the textual characteristics of the electrodes [15]. In addition, it was also reported that the incorporation of nanostructure into a Li host matrix exhibited an enhanced reversible capacity [8,16]. The coulombic efficiency (the ratio of the number of charges that enter the electrode to the number that can be extracted from the electrode) was more than 90% except for the initial few cycles, which suggests that the inserted Li ions were reversibly extracted. Figure 3c shows the current density dependence on the discharge capacities of the CoO*_x_*TF and CuNFs@CoO*_x_*at 0.15, 0.3, 0.6, 1.2, 2.5, and 3C-rates. The capacity of the CoO*_x_*TF decreased rapidly with increasing current density, which is consistent with previously reported results [15,17], whereas the CuNFs@CoO*_x_*was able to maintain 50% of its initial capacity even at 3C-rate. Such enhanced performance of the CuNFs@CoO*_x_*can be attributed to the improvement of the electrical conductivity of the CoO*_x_*by the embedded CuNF network, which creates an efficient electron percolation path between the current collector and the active material [8].

**Figure 4:**
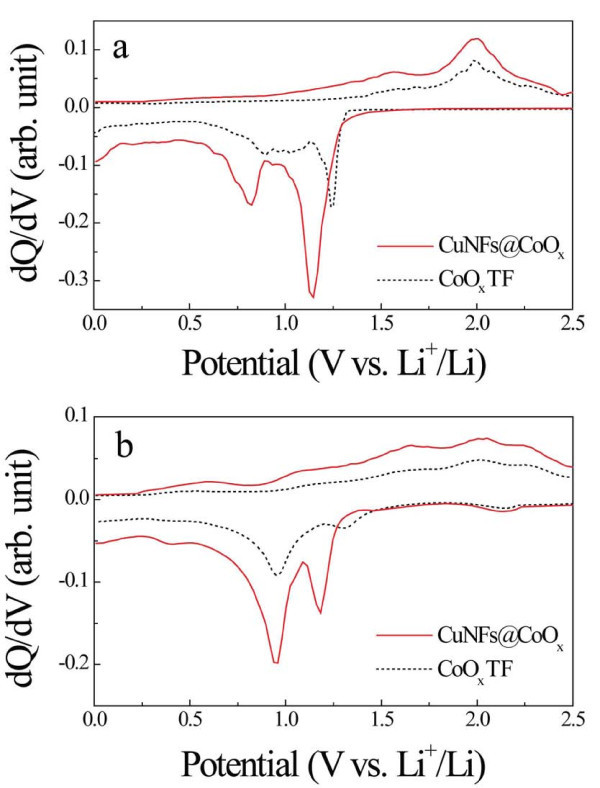
**Calculated differential capacity plots**. **(a) **the first cycle; **(b) **the tenth cycle of the CoO*_x_*TF and CuNFs@CoO*_x_*. The discharge process represents oxidation, while the charge process represents reduction processes.

To elucidate reason of the enhanced performance, the differential capacity was examined. Figure 4a, b was obtained from the first and tenth cycles, respectively. At the first cycle (Figure 4a), the intensity of the CuNFs@CoO*_x_*was larger than that of the CoO*_x_*TF, showing higher capacity and faster kinetics of the phase transformation. In Figure 4b, the decreased peak intensity and integral areas could be caused from the irreversible capacity due to the incomplete electrochemical reaction. Herein, it is interesting to find that some amount of previously formed Li_2_O phase would contribute to the capacity at tenth cycle. The formed Li_2_O has been generally reported to be electrochemically inactive. However, it was also reported that Li_2_O below 10 nm could be activated [1]. The activated Li_2_O can take place in the cyclic voltammetry results [18-20]. Two cathodic peaks at 0.82 and 1.15 V were observed in the first cycle in Figure 4a, but they were shifted to 0.95 and 1.18 V, respectively, in the subsequent cycles as shown in Figure 4b, indicating that the electrochemical reactions might be different from the first cycle. Thus, the electrochemical reactions in the CuNFs@CoO*_x_*composite with Li ions can involve the following steps [21-23]:

The first discharge process is an irreversible reaction of Co_3_O_4 _and Li, which forms metallic Co and Li_2_O phase. During the first charge process, the Co and Li_2_O forms CoO instead of Co_3_O_4 _owing to the similarity of oxygen lattice in the Li_2_O and CoO [24]. In the subsequent discharge/charge processes, the modified oxygen lattice is continuously preserved, indicating that the reaction of CoO with Li develops into reversible cycles.

**Figure 5:**
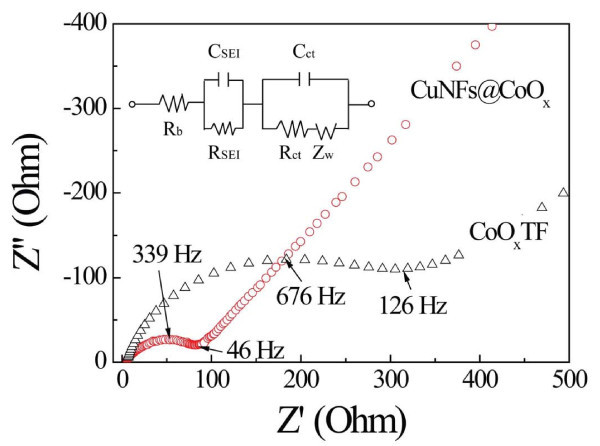
**AC impedance spectra for both samples**. The experimental results are presented as Nyquist plots by applying a sine wave with amplitude of 5 mV over the frequency range 100 kHz to 10 mHz, which were measured at *E *= 1.6 V (vs. Li^+^/Li) after the cycles.

In Figure 5, AC impedance measurements were performed to probe the kinetic factors contributing to the capacity and rate performance. The equivalent circuit analysis is based on a Randles equivalent circuit for an electrochemical system, in which *R*_b _is the bulk resistance, corresponding to the resistance value at the high-frequency intercept of the semicircle with the real axis [9,25]. *R*_ct _and *C*_ct _are the resistance of the charge-transfer and double-layer capacitance, respectively. The *R*_b _value of the CuNFs@CoO*_x_*was similar to that of the bare CoO*_x_*TF electrodes, whereas the *R*_ct _and *C*_ct _values for the CuNFs@CoO*_x_*were much smaller than those for CoO*_x_*TF. A considerable change in the sum of *R*_SEI _and *R*_ct _from 344 Ω was observed for CoO*_x_*TF to 96 Ω for CuNFs@CoO*_x_*, indicating an enhanced electrical conductivity arising from the composite, which implies that the charge transfer was significantly improved by the embedded CuNF network structure within the CoO*_x_*TF. This result confirmed that the embedded CuNF network could not only contribute to the high conductivity of the overall electrode, but also largely improve the electrochemical properties of CoO*_x_*during the cyclings.

### Mechanical stability

**Figure 6:**
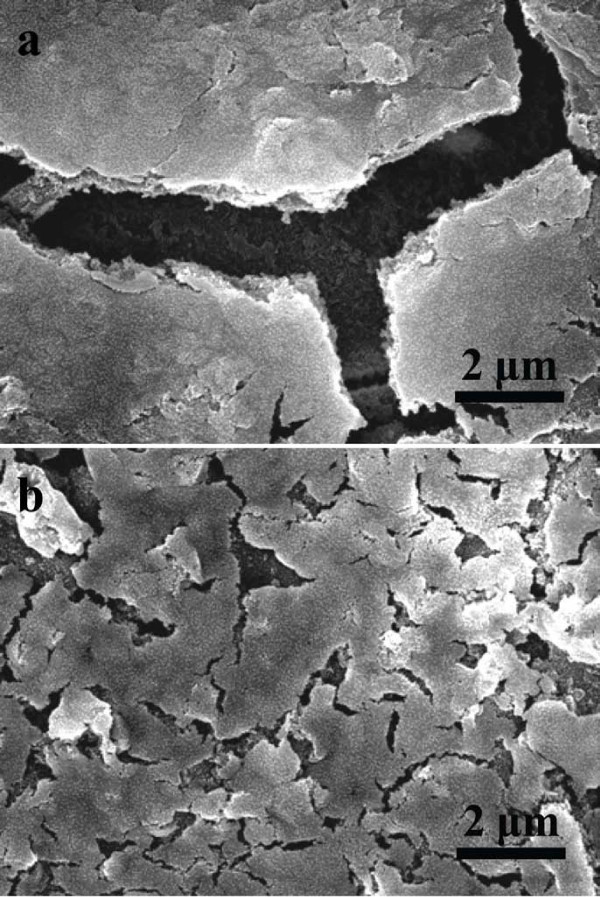
**FESEM images of the cycled CoO*_x_*electrodes.** (a) without and (b) with the Cu NFs after 30th cycle. The tested electrodes were disassembled and extracted from the Li-ion cell.

Figure 6 shows the FESEM images of the CoO*_x_*TF and CuNFs@CoO*_x_*after the 30th cycle. Two samples were disassembled after the electrochemical cycles in order to characterize the changes in the morphology. The CoO*_x_*TF appeared to experience serious cracking and crumbling, as shown in Figure 6a, while the CuNFs@CoO*_x_*seemed to remain fairly stable, as shown in Figure 6b. The CuNFs@CoO*_x_*maintained the integrity of the electrode with the current collector, suggesting the composite has the greater stress relaxation than the bare CoO*_x_*TF despite its higher capacity. This result implies that the embedded CuNF network significantly compensates the generated stress compared with the CoO*_x_*TF without the nanostructure. Thus, our results support the conclusion that embedded CuNF network nanostructures can significantly improve the capacity, rate performance, and mechanical stability of the CoO*_x_*electrode materials.

## Conclusions

A CuNFs@CoO*_x_*composite electrode was fabricated to serve as an anode for rechargeable Li-ion batteries. As an efficient Li-ion battery anode material, CuNFs@CoO*_x_*exhibited a higher capacity and rate performance than bare CoO*_x_*TF without CuNFs; the capacity at 0.15C was increased by ca. 30%, and the capacity was maintained above 50% even at 3C. These enhancements could be attributed to an increased number of reaction sites, facilitated charge transport, a decreased electrochemical double-layer capacitance, and facile stress relaxation by embedded CuNF network within the CoO*_x_*. Consequently, this CuNFs@CoO*_x_*composite structure can be a promising candidate for use in the electrodes of high-performance Li-ion batteries.

## Abbreviations

CuNFs@CoO*_x_*: copper nanofiber-networked cobalt oxide; CuNFs: copper nanofibers; CoO*_x_*TF: cobalt oxide thin-film; CoO*_x_*: cobalt oxide; FESEM: field emission scanning electron microscopy; PVP: polyvinylpyrrolidone; VO*_x_*: vanadium oxide; XPS: x-ray photoelectron spectroscopy; XRD: x-ray diffraction.

## Competing interests

The authors declare that they have no competing interests.

## Authors' contributions

SHN and WBK designed and drafted the study. SHN and YSK fabricated the electrode using the electrospinning and sputtering. HSS and JGK participated in the characterization. All authors read and approved the final manuscript.
